# A Simple Arrangement of a Fracture Box for the Leg

**Published:** 1853-06

**Authors:** Philip Kirwan


ART. IV.—*A Simple Arrangement of a Fracture Box for the Leg.* By PHILIP KIRWAN, M. D.

THE box is composed of two sides, a bottom, and foot board, made of inch pine lumber, and nailed or screwed solid together, and forming an inclined plane. Also a bed for the leg to rest on, made of thin but strong cotton factory cloth; which bed, with its appendages, might be said to be the only part new in the arrangement.

*Dimensions.*—The box I am about to describe will answer for legs of different sizes, and for both right or left, by a little manipulation, &c. The box is six inches deep, seven inches wide at one end, and four at the other; the length in the clear may be twenty to twenty-four inches from the foot board to the angle at the knee; the thigh portion, ten inches; the foot board may be ten or twelve inches long, and set obliquely forward; the sides are rounded on the upper edges; the inclination does not exceed three inches. The strip of cloth for the bed should be of such dimensions, that when one edge of the cloth is tacked with small tacks, along the upper and outer edge of the side of the box next the sound leg, and the other edge laps about an inch over the opposite side, the leg may sink in the box to within about an inch of the bottom. Both edges of the bed ought to be hemmed before being tacked, and pieces of strong tape, nine or ten inches long, sewed to the free edge of the bed or cloth, at a distance of about three inches apart, to be twined around small pegs or screws to be inserted along the under edge of the side of the box about half an inch up from the bottom edge, and at corresponding distances with the pieces of tape spoken of. The object of the tapes and pegs or screws is to support the bed on which the leg is placed, at a desired distance from the bottom of the box, and thereby admit the air to circulate freely about the leg, and also to relieve pressure of the leg or thigh at any part, by relaxing a tape opposite to the pressure, &c.

It may be asked, can extension be made if required ? I answer it can, by a little ingenuity on the part of the physician.

I have tested the above described box, and am led to believe that it possesses two or three important advantages to the patient. 1st. The facility with which pressure on any part of the leg or thigh can be relieved, without the least disturbance to the leg. 2d. The free access of air admitted around the leg, and thereby preventing no small amount of heat, swelling, and, consequently, pain. 3d. The evenness of the bed on which the leg rests, affording much ease, and but little occasion to relieve pressure of the leg. The leg may be made immovable in the box, if desired, with cotton batting, &c., and a roller around the box and leg.

